# MiR-204 reduces cisplatin resistance in non-small cell lung cancer through suppression of the caveolin-1/AKT/Bad pathway

**DOI:** 10.18632/aging.101907

**Published:** 2019-04-12

**Authors:** Gang Huang, Tianzheng Lou, Jiongwei Pan, Zaiting Ye, Zhangyong Yin, Lu Li, Wei Cheng, Zhuo Cao

**Affiliations:** 1 Department of Traditional Chinese Medicine, The Sixth Affiliated Hospital of Wenzhou Medical University, Lishui People’s Hospital, Lishui, Zhejiang 323000, China; 2 Department of Intensive Care Unit, The Sixth Affiliated Hospital of Wenzhou Medical University, Lishui People’s Hospital, Lishui, Zhejiang 323000, China; 3 Department of Respiratory, The Sixth Affiliated Hospital of Wenzhou Medical University, Lishui People’s Hospital, Lishui, Zhejiang 323000, China; 4 Department of Radiology, The Sixth Affiliated Hospital of Wenzhou Medical University, Lishui People’s Hospital, Lishui, Zhejiang 323000, China; 5 Department of Anesthesiology, Affiliated Hospital of Xuzhou Medical University, Jiangsu Province Key Laboratory of Anesthesiology and Center for Pain Research and Treatment, Xuzhou, Jiangsu 221002, China; 6 The People's Hospital of Kizilsu Kirghiz Autonomous Prefecture, Xinjiang 845350, China; * Equal contribution

**Keywords:** miR-204, caveolin-1, cisplatin, resistance, NSCLC

## Abstract

Non-small cell lung cancer (NSCLC) is the most common and lethal human malignant tumor worldwide. Platinum-based chemotherapy is still the mainstay of treatment for NSCLC. However, long-term chemotherapy usually induces serious drug resistance in NSCLC cells. Accordingly, treatment strategies that reverse the resistance of NSCLC cells against platinum-based drugs may have considerable clinical value. In the present study, we observed significant upregulation of CAV-1 expression and a significant decrease of miR-204 expression in cisplatin-resistant A549 (CR-A549) and cisplatin-resistant PC9 (CR-PC9) cells compared to their parental A549 and PC9 cells. Furthermore, we demonstrated that the downregulation of miR-204 expression was responsible for CAV-1 overexpression in these cisplatin-resistant NSCLC cells. We then found that enforced expression of miR-204 can resensitize CR-A549 and CR-PC9 cells to cisplatin-induced mitochondrial apoptosis through suppression of the caveolin-1/AKT/Bad pathway. We demonstrated that dysregulation of miR-204/caveolin-1 axis is an important mechanism for NSCLC cells to develop the chemoresistance.

## INTRODUCTION

Non-small cell lung cancer (NSCLC) is the leading cause of cancer-related deaths worldwide. Despite great advances in the diagnosis and treatment strategies used for NSCLC in recent decades, the prognosis of NSCLC remains poor, and the 5-year overall survival rate of NSCLC is still lower than 15% [1, 2]. Platinum-based chemotherapy remains a widely used first-line strategy for the treatment of NSCLC. However, the clinical response to platinum-based chemotherapy is limited because NSCLC cells usually develop resistance after the repeated use of platinum-based drugs. Ultimately, the drug resistance induces failure of chemotherapy and poor prognoses [3, 4].

Cisplatin is the most commonly used platinum-based chemotherapeutic drug, and it exhibits effective and broad-spectrum anti-tumor activity against multiple cancers, including NSCLC [5, 6]. As a platinum-based drug, cisplatin can cross-link with DNA and subsequently inhibit DNA replication and transcription and trigger the apoptosis pathway in NSCLC cells [7]. However, under the long-term use of cisplatin, cancer cells usually develop severe chemoresistance, which has become a major obstacle for the clinical use of cisplatin in patients with NSCLC [8–10]. Therefore, attenuation of the acquired chemoresistance to cisplatin in NSCLC treatment is an important clinical objective.

MicroRNAs (miRNAs) are endogenous and non-coding RNAs 19–25 nucleotides in length. As miRNAs regulate the expression of approximately 60% of human genes through binding to 3′ untranslated region (3′ UTR) of targeted mRNAs, they are involved in many physiological processes, including cell proliferation, differentiation, metastasis, metabolism, and apoptosis. Unfortunately, miRNAs are usually dysregulated in cancer cells [11–13]. It has been demonstrated that the aberrant expression of miRNAs is closely associated with tumorigenesis and cancer development. Moreover dysregulation of miRNAs is responsible for the induction of drug resistance in many cancers, including NSCLC. Therefore, correction of miRNA dysregulation may represent a potential strategy for reversing chemoresistance [14–16]. In this study, we investigated the role of miR-204 in reducing cisplatin resistance in NSCLC.

## RESULTS

### Cisplatin resistance of CR-A549 and CR-PC9

**Figure 1:**
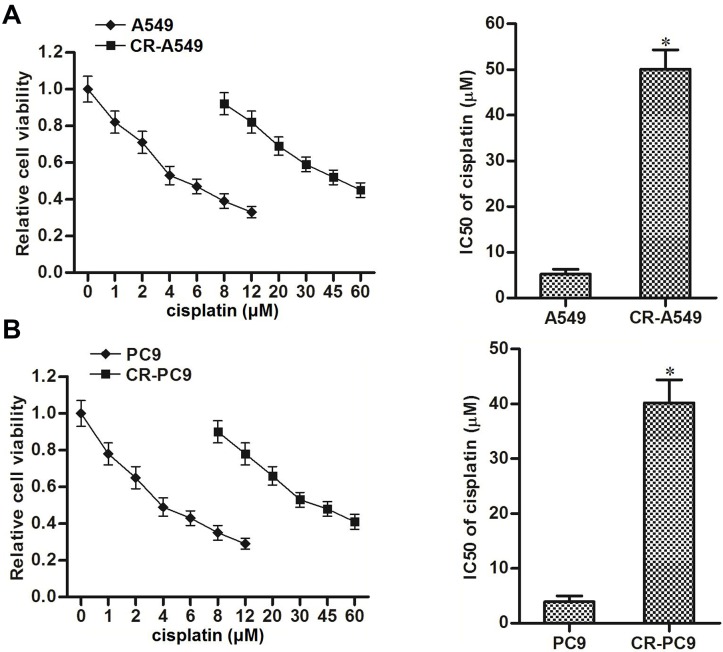
**Cisplatin resistance of CR-A549 and CR-PC9 cells.** (**A**) After treatment with different concentrations of cisplatin (0–60 μM), viability of A549 and CR-A549 cells was detected by using MTT assays. *P<0.05* vs.* A549 cells. (**B**) After treatment with different concentrations of cisplatin (0–60 μM), viability of PC9 and CR-PC9 cells was detected by using MTT assays. *P<0.05* vs.* PC9 cells.

To study the chemoresistance of NSCLC cells, cisplatin-resistant NSCLC models were established using A549 and PC9 cell lines (CR-A549 and CR-PC9). As shown in Figure 1A, MTT assays showed that CR-A549 cells exhibited significant cisplatin resistance compared to their parental A549 cells, and the IC50 value of cisplatin for CR-A549 increased 9.8-fold compared with that for A549. Similarly, the cisplatin IC50 value for CR-PC9 increased 12.4-fold compared with that for PC9 (Figure 1B). Accordingly, our established CR-A549 and CR-PC9 cells were cisplatin-resistant. In addition, because 8 μM cisplatin induced slight cytotoxicity against CR-A549 and CR-PC9 cells, we chose this concentration of cisplatin in the following experiments to explore the potential mechanisms of cisplatin-resistance formation in CR-A549 and CR-PC9.

### Upregulation of CAV-1 is essential for cisplatin resistance in NSCLC

**Figure 2:**
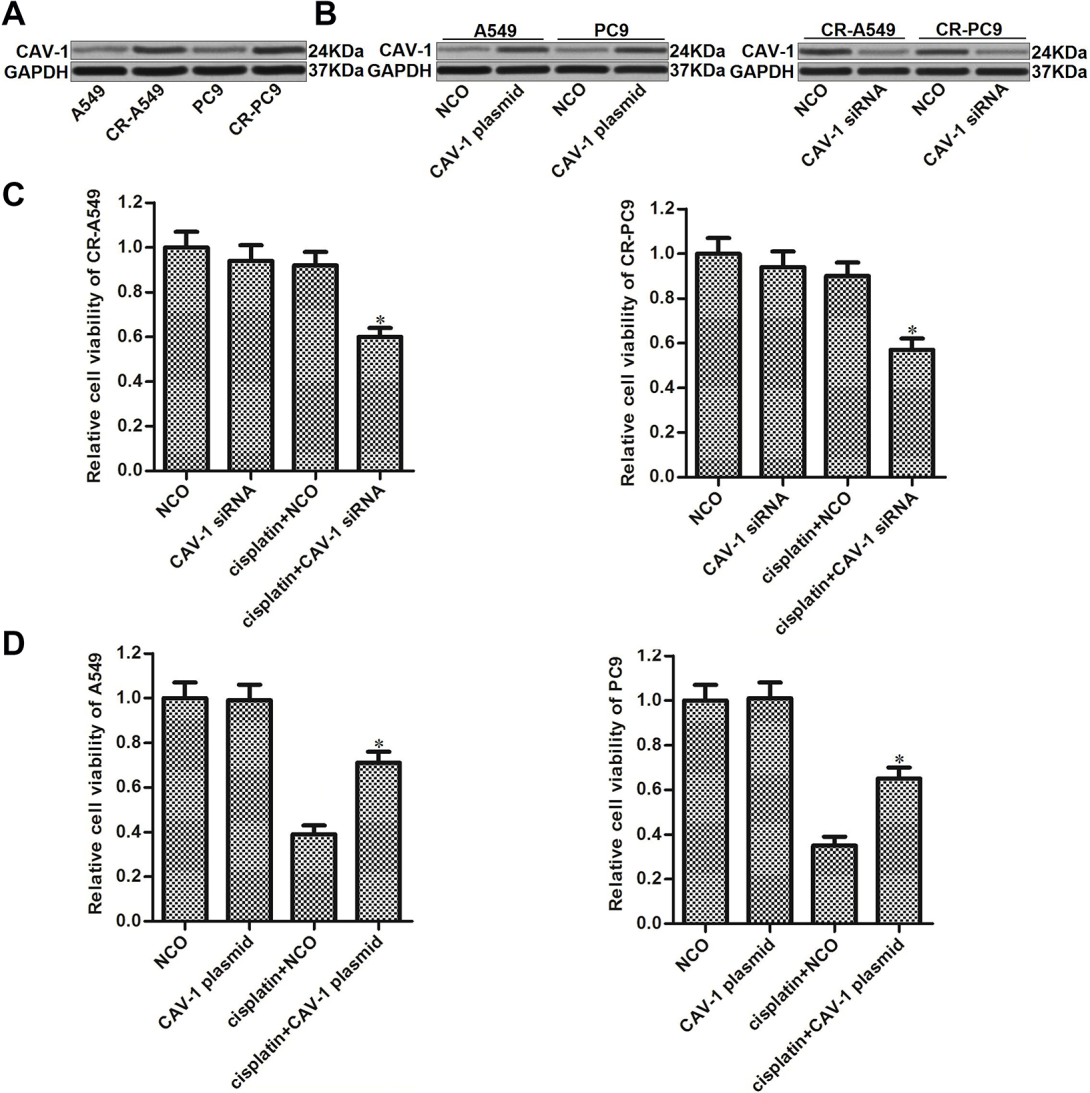
**Role of CAV-1 in regulating cisplatin sensitivity in NSCLC**. (**A**) Expression of CAV-1 in A549, CR-A549, PC9, and CR-PC9 cells was detected by western blot analysis. (**B**) Effect of the CAV-1 plasmid (2 μg/ml) and CAV-1 siRNA (50 pmol/ml) on the expression level of CAV-1 in A549, CR-A549, PC9, and CR-PC9 cells. (**C**) Effect of CAV-1 siRNA (50 pmol/ml) on the sensitivity of CR-A549 and CR-PC9 cells to cisplatin (8 μM) treatment. *P<0.05* vs.* Cisplatin+NCO group. (**D**) Effect of the CAV-1 plasmid (2 μg/ml) on the sensitivity of A549 and PC9 cells to cisplatin (8 μM) treatment. *P<0.05* vs.* Cisplatin+NCO group.

Upregulation of CAV-1 has been reported to contribute to multiple drug resistance in cancer cells [17–19]. We therefore investigated the potential role of CAV-1 in CR-A549 and CR-PC9. We found that the expression of CAV-1 in CR-A549 and CR-PC9 cells was higher than that in their parental A549 and PC9 cells, respectively (Figure 2A). To investigate whether the upregulation of CAV-1 was responsible for cisplatin resistance in these CR-A549 and CR-PC9 cells, we performed a loss-of-function assay using specific siRNA targeting CAV-1, and the transfection efficiency of CAV-1 siRNA is shown in Figure 2B. Interestingly, we found that the knockdown of CAV-1 significantly increased the cytotoxicity of cisplatin against CR-A549 and CR-PC9 (Figure 2C). On the other hand, enforced expression of CAV-1 in A549 and PC9 cells reduced the cytotoxicity of cisplatin against them (Figure 2D). Accordingly, the expression profile of CAV-1 was associated with cisplatin sensitivity in NSCLC cells. As the expression of CAV-1 was dysregulated in cisplatin-resistant NSCLC cells, the inhibition of CAV-1 attenuated the acquired cisplatin resistance in NSCLC.

### Upregulation of CAV-1 is induced by the decrease in miR-204 in NSCLC

**Figure 3:**
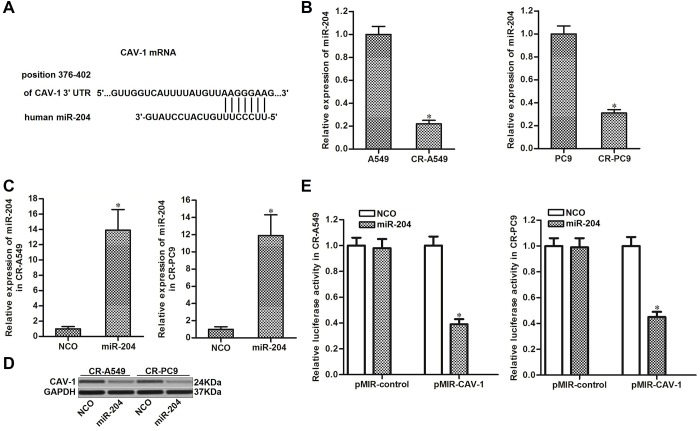
**miR-204 targets CAV-1 in NSCLC.** (**A**) Seed region of the CAV-1 3′ UTR paired with miR-204. (**B**) Expression of miR-204 in A549, CR-A549, PC9, and CR-PC9 cell lines. *P<0.05. (**C**) Transfection efficiency of miR-204 in CR-A549 and CR-PC9 cells. *P<0.05* vs.* NCO group. (**D**) Effect of miR-204 (50 pmol/ml) on the expression level of CAV-1 in CR-A549 and CR-PC9 cells. (**E**) Luciferase activities in CR-A549 and CR-PC9 cells were measured using the Dual-Luciferase Reporter Assay System. *P<0.05* vs.* NCO group.

We next investigated whether the overexpression of CAV-1 was caused by the dysregulation of miRNAs in CR-A549 and CR-PC9. Data from the public miRNA prediction databases TargetScan, miRanda, and PicTar showed that the *CAV-1* gene contains a seed region paired with miR-204 in the 3′ UTR of its mRNA (Figure 3A). miR-204 has been reported as a sensitizer that enhances the anti-tumor effect of chemotherapeutic drugs [20, 21]; thus, we focused on the relationship between miR-204 and CAV-1 in CR-A549 and CR-PC9 cells. As shown in Figure 3B, expression of miR-204 was decreased when the NSCLC cell lines became cisplatin-resistant. We therefore performed gain-of-function assays by transfection with miR-204 mimics (Figure 3C). Interestingly, transfection with miR-204 decreased the expression of CAV-1 in CR-A549 and CR-PC9 cells (Figure 3D). Furthermore, results of luciferase reporter assays showed that co-transfection with miR-204 was able to decrease the luciferase activities of the pMIR-plasmid carrying the caveolin-1 3′ UTR (Figure 3E). Thus, the upregulation of CAV-1 was induced by the decrease in miR-204 in cisplatin-resistant NSCLC cells.

### miR-204 resensitizes cisplatin-resistant NSCLC cells to cisplatin through the inhibition of CAV-1

**Figure 4:**
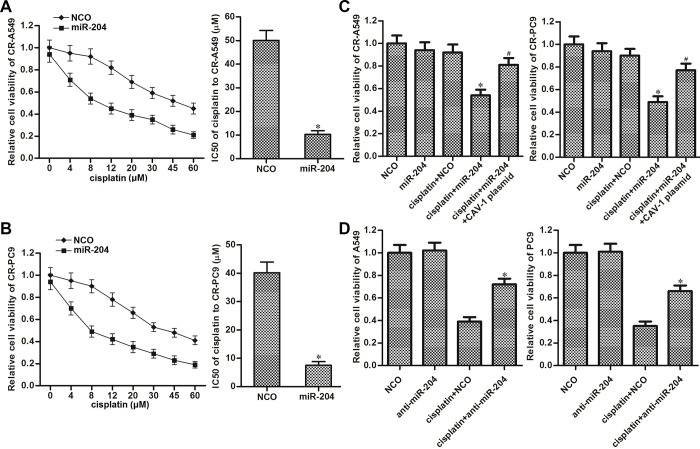
**Role of the miR-204/CAV-1 axis in regulating cisplatin sensitivity in NSCLC.** (**A**) Effect of miR-204 (50 pmol/ml) on the sensitivity of CR-A549 cells to cisplatin treatment (0–60 μM). *P<0.05* vs.* NCO group. (**B**) Effect of miR-204 (50 pmol/ml) on the sensitivity of CR-PC9 cells to cisplatin treatment (0–60 μM). *P<0.05* vs.* NCO group. (**C**) Effect of the CAV-1 plasmid (2 μg/ml) on protecting the CR-A549 and CR-PC9 cells that were co-treated with cisplatin (8 μM) and miR-204 (50 pmol/ml). *P<0.05* vs.* Cisplatin+NCO group. ^#^*P*<0.05* vs.* Cisplatin+miR-204 group. (**D**) Effect of the anti-miR-204 (50 pmol/ml) on protecting the A549 and PC9 cells that were co-treated with cisplatin (8 μM). *P<0.05* vs.* Cisplatin+NCO group.

We next investigated the role of the miR-204/CAV-1 axis in changing the cisplatin sensitivity in CR-A549 and CR-PC9 cells. As shown in Figure 4A, transfection with miR-204 decreased the cisplatin resistance in NSCLC, and the IC50 value of cisplatin for CR-A549 was reduced by 79.5% after miR-204 transfection. Similarly, the cisplatin IC50 value for CR-PC9 was reduced by 83.6% after transfection with miR-204 (Figure 4B). However, we found that enforced expression of CAV-1 “rescued” the CR-A549 and CR-PC9 cells that were co-treated with cisplatin and miR-204 (Figure 4C). These results indicated that miR-204 was able to resensitize the cisplatin-resistant NSCLC cells to cisplatin-induced cytotoxicity through the inhibition of CAV-1. Additionally, we also found that knockdown of miR-204 directly by using its antisense oligonucleotides (anti-miR-204) decreased the cytotoxicity of cisplatin to the parental A549 and PC9 cells (Figure 4D). This data emphasized the sensitization of miR-204 on cisplatin treatment in NSCLC.

### The miR-204/CAV-1 axis regulates the AKT/Bad pathway in cisplatin-resistant NSCLC cells

**Figure 5:**
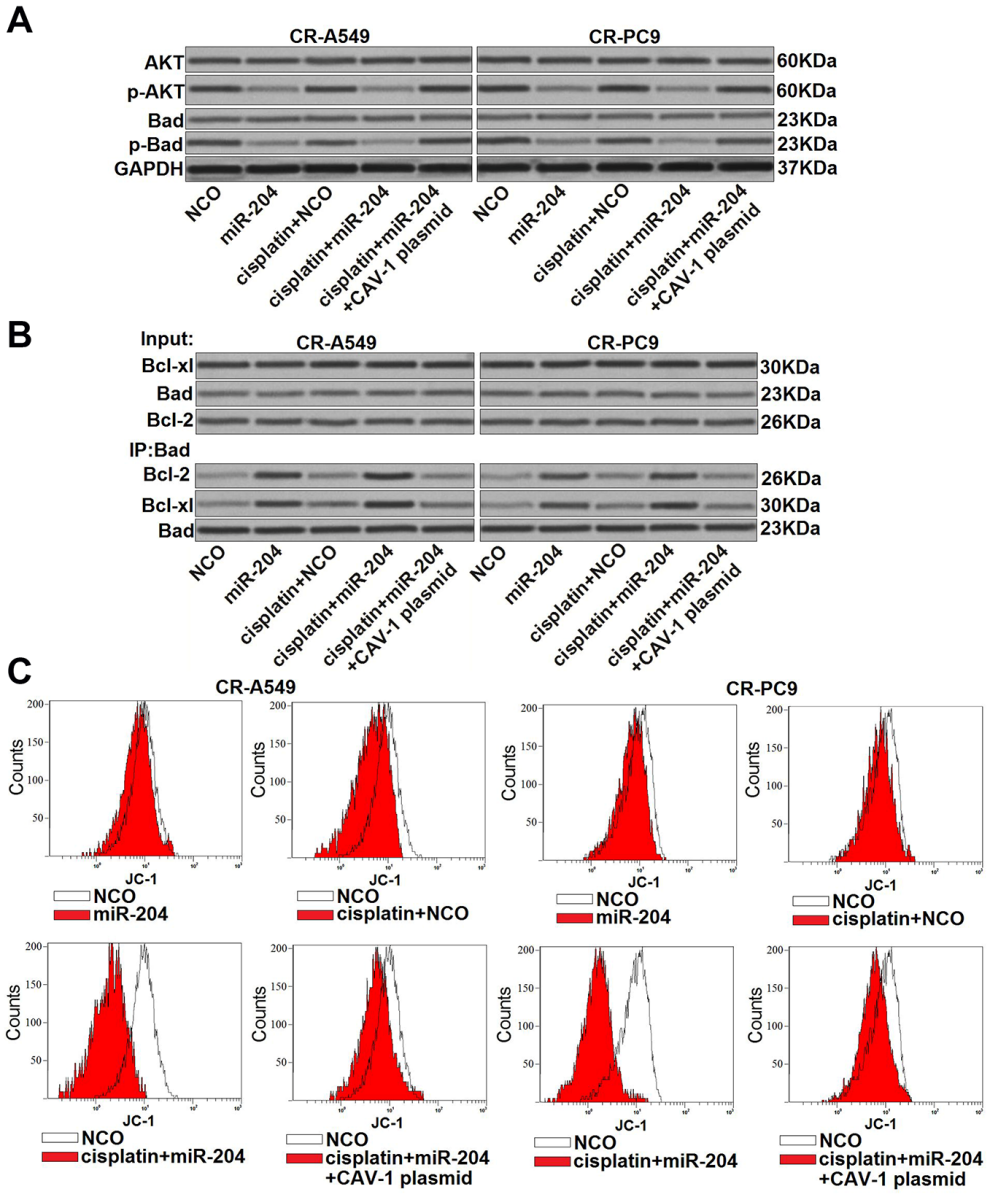
**miR-204/CAV-1 axis regulates the AKT/Bad pathway in cisplatin-resistant NSCLC cells.** (**A**) Effect of miR-204 (50 pmol/ml), cisplatin (8 μM), and the CVA-1 plasmid (2 μg/ml) on the phosphorylation of AKT and Bad in CR-A549 and CR-PC9 cells. (**B**) A co-immunoprecipitation assay was performed to evaluate interactions with Bad and Bcl-xl/Bcl-2 after treatment with miR-204 (50 pmol/ml), cisplatin (8 μM), and the CVA-1 plasmid (2 μg/ml). (**C**) Effect of miR-204 (50 pmol/ml), cisplatin (8 μM), and the CVA-1 plasmid (2 μg/ml) on the mitochondrial membrane potential (ΔΨ_m_) of CR-A549 and CR-PC9 cells.

CAV-1 is an upstream regulator of the AKT pathway, and the overexpression of caveolin-1 usually induces overactivation of AKT in cancer cells [22]. As Bad is one important substrate of AKT [23, 24], we next explored the role of the miR-204/CAV-1 axis in regulating the AKT/Bad pathway in CR-A549 and CR-PC9 cells. Western blot analysis showed that transfection with miR-204 decreased the phosphorylation of AKT and Bad in CR-A549 and CR-PC9 cells. However, enforced expression of CAV-1 using the CAV-1 plasmid inhibited the effect of miR-204 on AKT and Bad phosphorylation (Figure 5A). This indicated that the AKT/Bad pathway was regulated by the miR-204/CAV-1 axis in cisplatin-resistant NSCLC cells. Subsequently, we observed that miR-204 treatment increased the levels of the Bad-Bcl-2 and Bad-Bcl-xl heterodimers in CR-A549 and CR-PC9 cells (Figure 5B). Thus, we found that miR-204 attenuated the function of free Bcl-2 and Bcl-xl through the miR-204/CAV-1/Bad pathway. Because both Bcl-2 and Bcl-xl are anti-apoptotic proteins that inhibit mitochondrial apoptosis [25, 26], we next investigated the effect of cisplatin and miR-204 on ΔΨ_m_ for CR-A549 and CR- PC9 cells. We found that cisplatin alone induced a slight decrease in ΔΨ_m_ for CR-A549 and CR-PC9 cells. However, in combination with miR-204, there was an obvious promotion of the cisplatin-induced collapse of ΔΨ_m_ (Figure 5C). These results suggested that the miR-204/CAV-1 axis regulates the mitochondrial apoptosis of cisplatin-resistant NSCLC cells through the AKT/Bad/Bcl-2 (Bcl-xl) pathway.

### miR-204 enhanced cisplatin-induced mitochondrial apoptosis of cisplatin-resistant NSCLC cells

**Figure 6:**
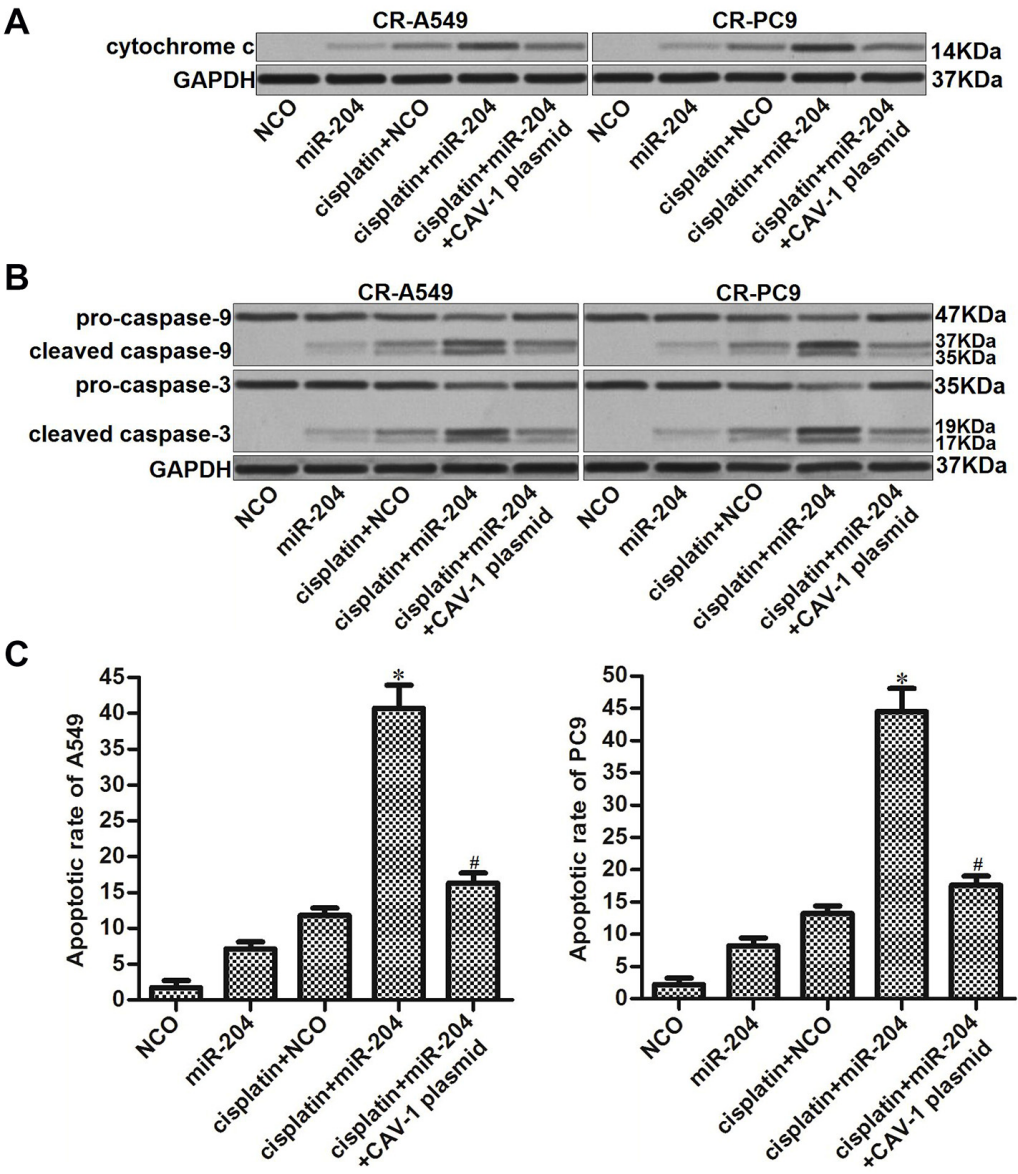
**miR-204/CAV-1 axis regulates the mitochondrial apoptosis pathway in cisplatin-resistant NSCLC cells.** (**A**) After mitochondria removal, the protein level of cytochrome c in cytosol was measured using western blot analysis. (**B**) Cleavage of caspase-9 and caspase-3 in CR-A549 and CR-PC9 cells was detected using western blot analysis. (**C**) Effect of miR-204 (50 pmol/ml), cisplatin (8 μM), and the CVA-1 plasmid (2 μg/ml) on the apoptotic rate of CR-A549 and CR-PC9 cells. *P<0.05* vs.* Cisplatin+NCO group. ^#^*P*<0.05* vs.* Cisplatin+miR-204 group.

The preceding results have indicated that miR-204 was able to increase the cisplatin-induced collapse of ΔΨ_m_ in CR-A549 and CR-PC9 cells. Accordingly, we next evaluated the effect of miR-204 on the cisplatin-induced mitochondrial apoptosis pathway. Western blot analysis showed that miR-204 transfection obviously increased the release of cytochrome c from mitochondria into the cytosol. However, enforced expression of CAV-1 decreased the level of cytochrome c in the cytosol of CR-A549 and CR-PC9 cells that were co-treated with cisplatin and miR-204 (Figure 6A). Moreover, we found that the combination of cisplatin and miR-204 induced obvious cleavage of caspase-9 and caspase-3, whereas transfection with the CAV-1 plasmid attenuated the activation of these caspases (Figure 6B). Accompanied by caspase cleavage, the combination of miR-204 and cisplatin induced a significantly higher rate of apoptosis compared to that in the cisplatin alone treatment group (Figure 6C). Thus, miR-204 can enhance the cisplatin-induced mitochondrial apoptosis of cisplatin-resistant NSCLC cells.

### Overexpression of miR-204 enhances the anti-tumor effect of cisplatin against cisplatin-resistant NSCLC in vivo

**Figure 7:**
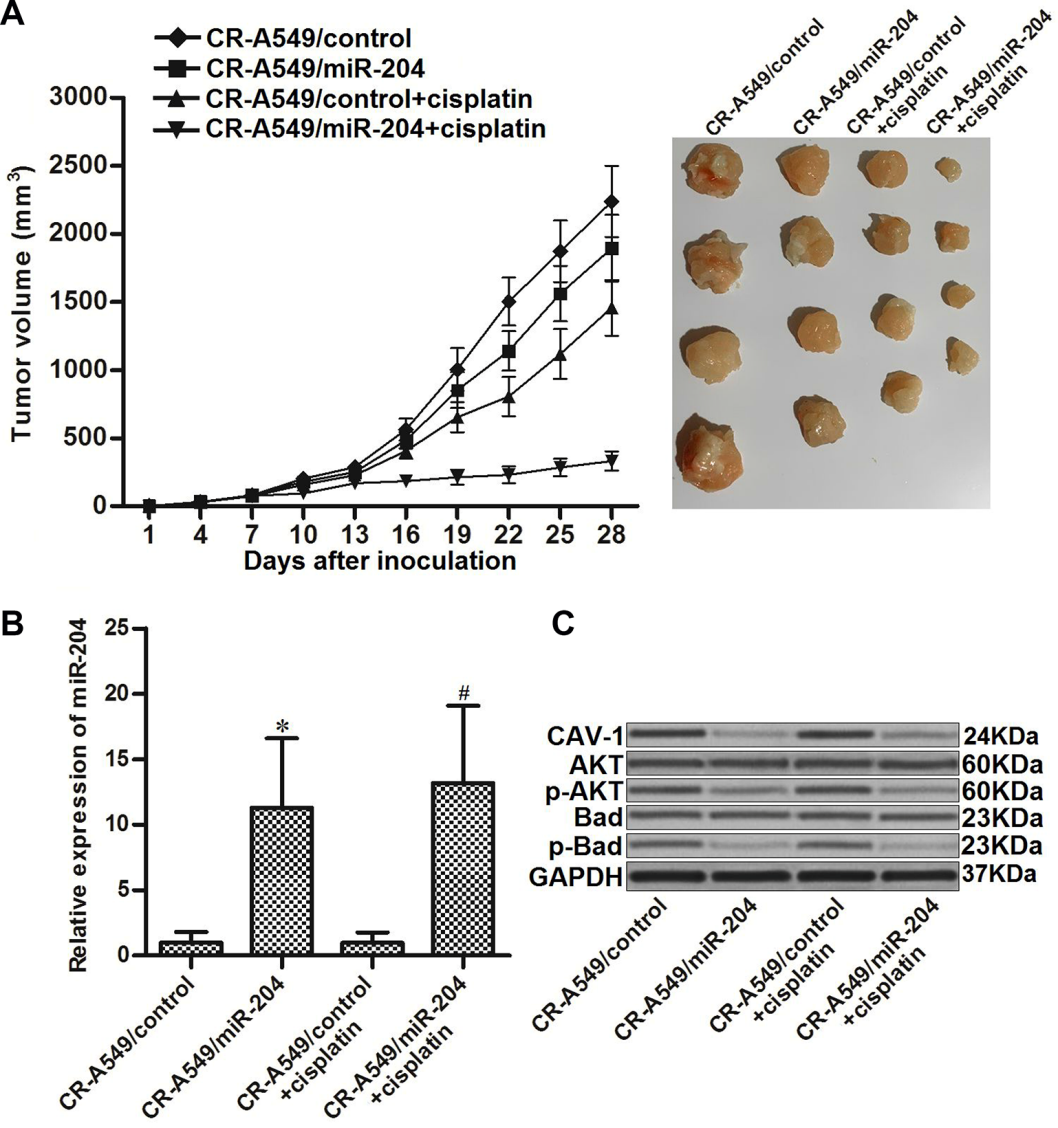
**miR-204 enhances the anti-tumor effect of cisplatin on cisplatin-resistant NSCLC in vivo.** (**A**) Nude mice were inoculated with CR-A549/control or CR-A549/miR-204 cells before treatment with cisplatin (5 mg/kg) twice a week. Tumor volumes were detected every three days until the experimental end-point (28 days post-injection). (**B**) Expression of miR-204 in tumor tissues was measured by qRT-PCR analysis. *P<0.05* vs.* CR-A549/control group. ^#^*P*<0.05* vs.* CR-A549/control+cisplatin group. (**C**) Expression of CAV-1 and phosphorylation of AKT and Bad were evaluated by western blot analysis.

To investigate the role of miR-204 in changing the cisplatin sensitivity of CR-A549 cells in vivo, we established an in vivo NSCLC model using CR-A549/control and CR-A549/miR-204 cells in mice. As shown in Figure 7A, we found that miR-204 can enhance the effect of cisplatin on inhibiting tumor growth of CR-A549 cells in mice. This indicated that miR-204-overexpressing CR-A549 cells were more sensitive to cisplatin treatment compared to the control CR-A549 cells. After purification of tumor tissues, we observed an overexpression of miR-204 in CR-A549/miR-204 tumors (Figure 7B). In contrast, the expression of CAV-1 was downregulated in these CR-A549/miR-204 tumors. In addition, the CR-A549/miR-204 tumors exhibited lower levels of AKT and Bad phosphorylation compared with those in the CR-A549/control tumors (Figure 7C). Together, these results suggested that the CAV-1/AKT/Bad pathway has a role in reducing cisplatin resistance in NSCLC in vivo.

## DISCUSSION

Although cisplatin-based chemotherapy is the first-line treatment for NSCLC, development of acquired resistance against cisplatin is still a substantial difficulty during the course of chemotherapy [27]. Accordingly, mechanisms that determine cisplatin sensitivity in NSCLC must be identified to increase the effectiveness of cisplatin-based treatment. A recent series of studies have shown that CAV-1 is usually overexpressed and is responsible for chemoresistance in cancer cells. Cav-1 is a major structural component of the caveolae, which is located along plasma membrane invaginations and is involved in cell adhesion and signal transduction [28]. In cancer cells, CAV-1 functions as an anti-apoptotic protein that promotes cell survival [29, 30]. Moreover, studies have shown that the overexpression of CAV-1 is usually found in multidrug-resistant tumor cells and indicates poor prognosis in cancer patients [18, 19, 31]. Thus, CAV-1 is a potential target for improving chemotherapy.

In this study, we established cisplatin-resistant NSCLC models in A549 and PC9 NSCLC cell lines. We found that CAV-1 expression was increased when NSCLC became cisplatin-resistant. We therefore focused on the role of CAV-1 in regulating the sensitivity of NSCLC cells to cisplatin. Interestingly, we found that cisplatin resistance of CR-A549 and CR-PC9 cells was obviously reduced after CAV-1 knockdown. On the other hand, increased expression of CAV-1 decreased the chemosensitivity of cisplatin to that of the standard A549 and PC9 NSCLC cells. These data indicated the effect of CAV-1 on the development of acquired resistance against cisplatin in NSCLC.

Recent studies have indicated that the dysregulation of miRNAs is responsible for the development of chemoresistance [32]. Among these dysregulated miRNAs, miR-204 is usually downregulated in multiple cancers, including NSCLC. Moreover, miR-204 usually functions as a tumor suppressor in cancers. For example, miR-204 targets RAB22A to inhibit the proliferation and invasion of colorectal cancer cells. Decreased expression of miR-204 is associated with poor prognosis in patients with breast cancer In NSCLC, miR-204 has been found to inhibit metastasis of NSCLC through the suppression of NUAK1 [21, 33, 34]. Therefore, the overexpression of miR-204 may represent a potential strategy for treatment of NSCLC.

In this study, we observed a significant decrease of miR-204 expression in cisplatin-resistant NSCLC cells compared to the cisplatin-sensitive cells. Furthermore, we demonstrated that the overexpression of CAV-1 was induced by the absence of miR-204 in the cisplatin-resistant NSCLC cells. We found that recovery of miR-204 expression was able to reduce the cisplatin-resistance of NSCLC cells through the suppression of CAV-1, both in vitro and in vivo. Thus, the combination of cisplatin and miR-204 is a potential approach to attenuate the chemoresistance of NSCLC cells against cisplatin.

Bad is a pro-apoptotic protein belonging to the Bcl-2 family. Non-phosphorylated Bad can inactivate the anti-apoptotic proteins Bcl-2 and Bcl-xl through conjugation with them. However, phosphorylated Bad loses its ability to interact with Bcl-2 and Bcl-xl [35, 36]. In our mechanistic study, we found that the miR-204/CAV-1 axis inhibited the phosphorylation of AKT and thus decreased the cellular level of phosphorylated Bad. We then found that the overexpression of miR-204 in cisplatin-resistant NSCLC cells increased the cellular level of both Bad-Bcl-heterodimers and Bad-Bcl-xl heterodimers through the suppression of CAV-1. We then demonstrated that recovery of miR-204 resensitized the cisplatin-resistant NSCLC cells to cisplatin-induced mitochondrial apoptosis via the caveolin-1/AKT/Bad pathway.

## MATERIALS AND METHODS

### Cell culture

Human NSCLC cell lines A549 and PC9 were obtained from the American Type Culture Collection (Manassas, VA) and cultured in Dulbecco’s modified Eagle’s medium (DMEM; Gibco, Gaithersburg, MD) supplemented with 10% fetal bovine serum (Gibco). For the establishment of cisplatin-resistant NSCLC models, A549 and PC9 cells were exposed to increasing concentrations of cisplatin (Sigma-Aldrich; Merck KGaA, Darmstadt, Germany). Briefly, the A549 and PC9 cells were initially treated with 0.2 μM cisplatin for 1 month. Subsequently, cisplatin concentrations in the culture medium were increased every 2 weeks by 0.1 μM up to a final concentration of 2 μM. The established cisplatin-resistant A549 and PC9 cells were labeled CR-A549 and CR-PC9, respectively. To eliminate the influence of residual cisplatin in the culture medium, the CR-A549 and CR-PC9 cells were cultured in cisplatin-free DMEM for 2 weeks prior to the experiments. Cells were maintained in an incubator under 5% CO_2_ at 37°C.

### Quantitative reverse transcription real-time PCR

Relative expression of miR-204 was measured by using quantitative reverse transcription real-time PCR (qRT-PCR) analysis. Briefly, total RNA was extracted from A549, PC9, CR-A549, and CR-PC9 cells using TRIzol^®^ reagent (Invitrogen; Thermo Fisher Scientific, Inc., Waltham, MA). Subsequently, reverse transcription of total RNA was performed using a stem-loop RT primer (5′-CTCAACTGGTGTCGTGGAGTCGGCAATTCAGTTGAGCATAGGAT-3′, RiboBio, Guangzhou, China) and the PrimeScript RT reagent kit according to the manufacturer’s protocol (Takara Bio, Inc., Otsu, Japan) followed by PCR amplification of miR-204 using SYBR^®^ Premix Ex Taq II reagent (Takara Bio, Inc.). The relative expression of miR-204 was normalized to U6 snRNA expression levels and determined according to 2^-△△Ct^ analysis [37]. The sequences of miR-204 and U6 amplification primers are as follows: miR-204 forward primer, 5′-ACACTCCAGCTGGGTTCCCTTTGTCAT-3′; miR-204 reverse primer, 5′-TGGTGTCGTGGAGTCG-3′; U6 forward primer, 5′-CTCGCTTCGGCAGCACA-3′; U6 reverse primer, 5′-AACGCTTCACGAATTTGCGT-3′.

### Cell transfection

Recombinant pcDNA3.1 plasmids carrying the caveolin-1 (CAV-1) open reading frame (2 μg/ml), hsa-miR-204 mimic (miR-204, GenePharma Co. Ltd, Shanghai, China; 50 pmol/ml), hsa-miR-204 antisense oligonucleotides (anti-miR-204, GenePharma Co. Ltd; 50 pmol/ml), negative control oligonucleotide (GenePharma Co. Ltd; 50 pmol/ml), and CAV-1 small interfering RNA (CAV-1 siRNA, GenePharma Co. Ltd; 50 pmol/ml) were transfected into cells by using Lipofectamine 2000 (Invitrogen) according to the manufacturer’s instructions. Twenty-four hours after transfection, cells were collected for the following experiments.

### Luciferase reporter assay

The *CAV-1* 3′ UTR fragment containing the predicted miR-204 binding site was amplified and inserted downstream of the firefly luciferase gene in the pMIR-REPORT™ miRNA Expression Reporter Vector System (Thermo Fisher Scientific, Inc.). The recombinant plasmid was named pMIR-CAV-1. To perform the luciferase reporter assay, pMIR-CAV-1 (or the empty pMIR vector, pMIR-control; 2 μg/ml), *Renilla* luciferase pRL-TK plasmid (Promega, Madison, WI; 100 ng/ml), and miR-204 (50 pmol/ml) were co-transfected into cells using Lipofectamine 2000. Luciferase activities were measured 48 h after incubation by using the Dual-Luciferase Reporter assay system (Promega) according to the manufacturer’s instructions.

### Cell viability assay

MTT assays were performed to detect cell viability. Transfected cells were seeded in 96-well plates at a density of 5 × 10^3^ cells per well overnight at 37°C. After treatment with different concentrations of cisplatin for 48 h, 20 μl of 5 mg/ml MTT reagent (Sigma-Aldrich, St. Louis, MO) was added to each well for another 4 h of incubation. Cells were then suspended in 150 μl of dimethyl sulfoxide before detection of their absorbance at 570 nm by using an enzyme-linked immunosorbent assay microplate reader (Sunrise Microplate Reader, TECAN, Männedorf, Switzerland). The half maximal inhibitory concentration (IC50) of cisplatin was inferred from the cell viability curve.

### Mitochondria removal

To detect the release of cytochrome c from mitochondria into cytosol, cellular mitochondria were removed using the Mitochondria/Cytosol Fraction Kit (BioVision, Milpitas, CA) according to the manufacturer’s directions. The protein level of cytochrome c in cytosol was measured by western blot analysis.

### Co-immunoprecipitation

To detect interactions with Bad and Bcl-xl, a co-immunoprecipitation assay was performed. Briefly, cells were collected and lysed with cold RIPA lysis buffer (Cell Signaling Technology, Danvers, MA) for 15 min at 4°C. The supernatant was collected, to which primary Bad antibody was added (Cell Signaling Technology). After overnight incubation, protein A/G plus-Agarose beads (Santa Cruz Biotechnology, Santa Cruz, CA) were added and shaken on a horizontal shaker for 1 h at 4°C. Immunoprecipitated pellets were then collected and washed with cold RIPA lysis buffer. Finally, the immunoprecipitated pellets were mixed with the sodium dodecyl sulfate polyacrylamide loading buffer and boiled to remove the beads.

### Western blot analysis

Cells were collected and lysed with cold RIPA lysis buffer. Subsequently, the extracted proteins (50 µg) were separated by 12% sodium dodecyl sulfate polyacrylamide gel electrophoresis and transferred to a polyvinylidene fluoride membrane (EMD Millipore, Billerica, MA). The membrane was then probed with primary antibodies against caveolin-1 (Cat No. #3267), AKT (Cat No. #4685), phosphorylated (p)-AKT (Cat No. #4060), Bad (Cat No. #9292), p-Bad (Cat No. #9291), Bcl-xl (Cat No. #2764), Bcl-2 (Cat No. #4223), cytochrome c (Cat No. #4280), caspase-9 (Cat No. #9502), caspase-3 (Cat No. #9662), and GAPDH (Cat No. #5174) (Cell Signaling Technology) at 4°C overnight. Subsequently, the membrane was washed and probed with horseradish peroxidase-conjugated goat anti-rabbit immunoglobulin G (Cell Signaling Technology). Protein bands were detected using an enhanced chemiluminescent substrate (Thermo Fisher Scientific, Inc.).

### Mitochondrial membrane potential and apoptosis detection

For detection of the mitochondrial membrane potential (ΔΨ_m_), cells were collected and stained with JC-1 (Molecular Probes; Thermo Fisher Scientific, Inc.) as an indicator [38] according to the manufacturer’s instructions. To measure the apoptotic rate, cells were collected and stained with annexin V/propidium iodide (Sigma-Aldrich) for 15 min in the dark at room temperature. ΔΨ_m_ and cell apoptosis were analyzed by flow cytometry.

### Tumor growth in nude mice

To evaluate the role of miR-204 in regulating the cisplatin sensitivity of CR-A549 in vivo, a stable CR-A549 cell line overexpressing miR-204 was generated. Briefly, a recombinant lentivirus that contains the miR-204 precursor sequence was obtained from Shanghai Genechem Co., Ltd. (Shanghai, China). Then, 1 × 10^4^ CR-A549 cells were transfected with 5 × 10^5^ transducing units of lentivirus, and the cells (CR-A549/miR-204) were selected with 1 μg/ml puromycin for 2 weeks. An equal number (5 × 10^6^) of CR-A549/miR-204 or lentivirus-control transfected CR-A549 (CR-A549/control) cells was harvested and washed. For the tumorigenesis assay, two groups of mice were subcutaneously injected with the CR-A549/control (for the CR-A549/control and CR-A549/control + cisplatin groups, respectively), and the two groups of mice were subcutaneously injected with CR-A549/miR-204 cells (for CR-A549/miR-204 and CR-A549/miR-204 + cisplatin groups, respectively). The tumor volume (V) was calculated according to the following formula: length × (width^2^)/2. In the CR-A549/control + cisplatin and CR-A549/miR-204 + cisplatin groups, the mice received the cisplatin treatment i.p. twice a week (5 mg/kg) until the mice were sacrificed 28 days post-injection. All animal procedures and experimental protocols were approved by the Animal Care Committee of The Sixth Affiliated Hospital of Wenzhou Medical University/Lishui People’s Hospital.

### Statistical analysis

Data are presented as the mean ± standard deviation obtained from three independent experiments. For the comparative analysis, two-tailed Student’s *t*-tests were used to estimate the statistical differences between two groups. One-way analysis of variance and Bonferroni’s post-hoc tests were used to determine the differences among the groups. Statistical analyses were performed using SPSS 15.0 software (SPSS Inc., Chicago, IL). *P*<0.05 was considered to indicate a statistically significant difference.

## CONCLUSIONS

We demonstrated that the mechanism by which miR-204 reduced the cisplatin resistance in NSCLC was dependent on the caveolin-1/AKT/Bad pathway. It indicated that dysregulation of miR-204/caveolin-1 axis is an important mechanism for NSCLC cells to develop the chemoresistance.
